# Chemical Surface Modification Tolerance of Primary and Immortalized Macrophages and Stem Cells

**DOI:** 10.1002/cbic.202500185

**Published:** 2025-11-13

**Authors:** Kyle J. Winters, Yacun A. Shen, Emmanuel F. Rivera-Iglesias, Jeffrey D. Cullen, Bishnu P. Joshi, Michelle E. Farkas

**Affiliations:** Department of Chemistry, University of Massachusetts Amherst, 710 North Pleasant Street, Amherst, Massachusetts 01003, USA

**Keywords:** bioconjugation, cellular engineering, chemotaxis, hydrazide, NHS ester

## Abstract

Cells are being utilized across various applications, including as self-regenerating materials, imaging and/or therapeutic entities, and delivery vehicles, and have the potential to do more. In biological and medical applications, specific cell types, including macrophages and mesenchymal stem cells (MSCs), have often been used on account of their recruitment to disease sites and desirable biodistribution properties. Typically, delivery applications involve the internalization of substrates within the cell, however, this approach presents drawbacks and is not amenable to some uses. Alternatively, chemically modifying cell surfaces using the toolbox of biocompatible chemistries has been broadly applied, but with few direct comparisons, including regarding assessment(s) of effects on the cells. In this work, we sought to compare commonly utilized N-hydroxysuccinimide ester and hydrazide-based conjugations to immortalized and primary macrophages and MSCs. We incorporated both small molecules and avidin proteins using each approach, finding that cargo size plays a substantial role in modifications. Overall, conjugations were well-tolerated by primary and immortalized macrophages and MSCs; we observed no major impacts on viability and chemotactic response, but found some slight changes and trends depending on cell and modification type. This foundational work directly comparing the results and effects of multiple conjugation strategies across different cell types will benefit their use across a variety of applications.

## 1. Introduction

The use of modified T cell infusions as cancer immunotherapies in patients has brought to light the potential of cell-based agents. Beyond this, multiple cell types have been used and/or studied in areas such as therapy and drug delivery,^[1,2]^ regenerative medicine,^[3]^ diagnostics,^[4]^ and sensors (for both medical monitoring and environmental analyses).^[5]^ In immunotherapy, engineered cells such as T cells are designed to enhance the immune response, targeting and eliminating cancer cells or infections, as exemplified by CAR-T therapy.^[1]^ Cell-based therapies utilizing autologous cells have also demonstrated significant promise in accelerating wound healing and reducing scarring or inflammation.^[3,6]^ In diagnostics, cellular biosensors leverage the natural and/or engineered abilities of whole cells to sense and respond to environmental stimuli, such as chemicals, toxins, or biomolecules.^[5,7]^ Also notably, in the realm of drug delivery, cells are utilized as biological carriers to deliver therapeutic agents directly to target tissues.^[8]^ Modifying cells allows us to both enhance and/or alter innate cellular characteristics as well as install advantageous and/or useful functionalities that are not naturally present. For example, the specificity of cells can be improved by incorporation of antibodies onto the cell surface or addition of fluorescent/radiolabeled cargo can allow for tracking within a system.^[9,10]^

Current methods for cell modification primarily involve one of two strategies: internalization or cell surface attachment. In internalization, cells engulf or are altered to incorporate molecules or other entities (e.g., therapeutic agents, diagnostic materials, or functional biomolecules), into their intracellular environment. While a significant number of studies have utilized this approach,^[11–14]^ it often poses obstacles including limited control over uptake and release of payloads, which may also be subject to degradation within endosomal compartments and induce cytotoxic effects on the carrier cells. Alternatively, genetic or chemical modifications of cell surfaces, resulting in the exterior display of moieties have also been utilized in varied applications, including improvement of cellular therapeutics,^[15–17]^ delivery,^[18]^ and targeting.^[19]^ Multiple cell types have been utilized in this manner, including macrophages and stem cells, which are the focus of the work presented here. Cell membrane engineering can be broadly categorized into two types: noncovalent and covalent modifications. Noncovalent modifications rely on weak, reversible interactions between functional molecules and the cell membrane, and offer flexibility and minimal disruption to the cell’s native structure and function.^[20]^ However, a primary drawback is their susceptibility to shear stress, which can compromise the persistence on the cell surface.^[21]^ As a result, noncovalent interactions typically lack the robustness required for long-term applications.^[21]^ Further, ensuring uniform labeling of the surface is a challenge. Covalent conjugations can circumvent these issues, but any methods used must be performed at or around physiological temperature, pH, ionic strength, and osmolality to result in minimal alterations to the cells. Chemical modifications at the cell membrane can exploit endogenously occurring functionalities or utilize non-native moieties that are installed or result from the conversion of an existing one. Cell surfaces have been altered for the attachment of different entities like imaging agents,^[22]^ nanoparticles,^[23,24]^ antibodies,^[25,26]^ DNA-based probes^[27–29]^ and targeting agents,^[17]^ polyethylene glycol (PEG) chains,^[16]^ and drug molecules.^[30,31]^

Among functional groups present, amines and thiols present excellent handles to functionalize the cell membrane. The most commonly used strategy for the covalent modification of exposed lysine side chain amine (-NH_2_) groups involves amide bond formation with an N-hydroxysuccinimide (NHS) ester.^[32,33]^ The use of this reaction facilitates stable covalent conjugations and ensures reliable and robust functionalization. Studies have demonstrated that appendages attached using this method remain intact in sufficient concentrations over several days, even during cell proliferation.^[34]^ This approach has been widely applied to conjugate molecules, such as polyethylene glycol PEG-DNA conjugates for cellular capture,^[35]^ pH nanosensors,^[36]^ hyperbranched polyglycerols for immune recognition reduction,^[37]^ or biotin for studying intercellular interactions.^[38]^ Surface thiol groups (-SH), present either as free thiols or as disulfide bridges are used in reactions with maleimide derivatives to form thioether bonds.^[33]^ The reactions of cell surface thiols with maleimide-modified liposomes and nanoparticles have emerged as tools for enhancing cellular interactions and uptake,^[39]^ as well as for bioanalysis and nanomedicine applications.^[40]^ However, this approach is not as frequently used as NHS-based modifications. Both of these methodologies are not without drawbacks. NHS-ester-bearing substrates can hydrolyze in the presence of water, undergo nonspecific reactions with other nucleophilic species, and can possess limited solubility without the use of organic co-solvents. Additionally, maleimide-thiol systems suffer from potential side product formation as well as degradation of adducts due to instability. In addition to the direct modifications of proteins, functionalizations and conjugations at carbohydrate/glycan moieties are also of interest and broadly used. A notable example is sialic acid, a terminal glycan ubiquitously present on cell surfaces.^[41]^ While it lacks a naturally reactive handle, two strategies for modification can be used. One approach involves oxidizing the vicinal diols on sialic acid residues with mild periodate that generates reactive aldehyde groups. The aldehydes can then react with hydrazide-conjugates to form hydrazone linkages.^[41]^ The second approach is the introduction of and bioorthogonal conjugation to non-natural azido-sugars.^[42,43]^ However, based on the fact that the metabolic incorporation of azido sugars can take a significant amount of time (on the order of days), a more efficient means of modifying carbohydrates is typically needed.^[43]^

As an alternative to direct conjugation of small molecules, incorporation of biotin on the cell surface via an array of strategies (including the ones described above) is also possible. Once biotin has been introduced it is capable of acting as a molecular “bridge” to attach various cargoes through the highly stable streptavidin–biotin interaction. This approach takes advantage of the exceptionally strong, noncovalent binding affinity between biotin (a small molecule) and streptavidin (a protein), recognized as one of the strongest known biological interactions. The streptavidin–biotin system enhances the functionality of molecules attached for diverse applications in cancer vaccines,^[44]^ cancer drug delivery,^[45–47]^ and controlling cell–cell interactions.^[48]^

**Figure 1.**
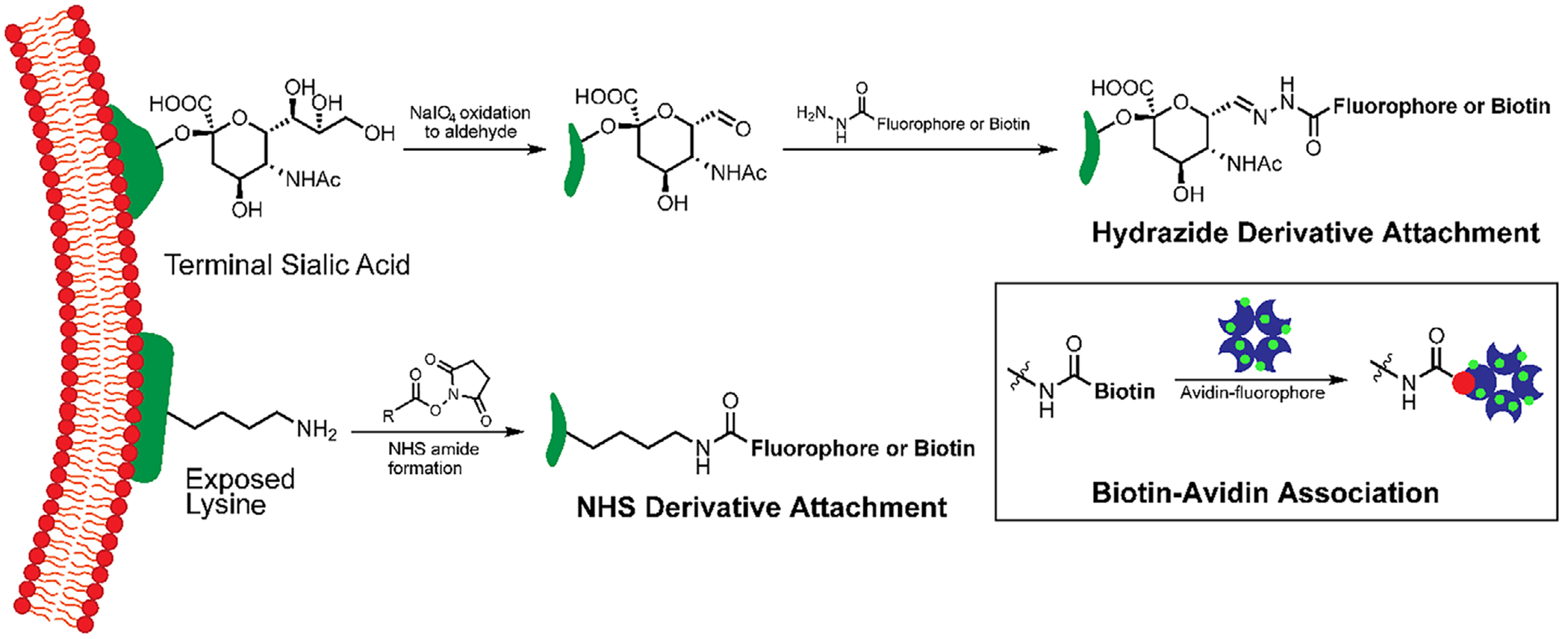
Overview of chemical reactions used to modify cell surfaces. Oxidation of terminal sialic acid residues with sodium periodate (NaIO_4_) is followed by a reaction with hydrazide-bearing molecules to yield hydrazone linkages (above). Reactions of free amines on lysines with NHS esters yield amide linkages (below). These reactions can be used to directly conjugate fluorophores or biotin moieties to the cell surfaces. Following biotinylation, avidin-FITC can be introduced and associated with surface-conjugated biotins (red orb represents biotin; inset).

While covalent cell membrane modifications have been previously performed in various cell types with cargos of differing sizes, a direct comparison among cell types, conjugation methods, and cargo sizes is currently lacking and would provide critical information for the modifications of cells in various contexts. Our previous work successfully demonstrated the modification of cell surface molecules on immortalized macrophages (RAW 264.7 cells) using NHS- and metabolically incorporated azide-mediated conjugations, with little to no detrimental effects on polarization/ phagocytosis, and chemotactic migration.^[22]^ On account of the importance of primary cells and mesenchymal stem cells (MSCs) to the applications described above, we deemed it critical to assess the effects of modifications on these cell types. For optimization, RAW 264.7 cells and immortalized human telomerase reverse transcriptase (hTERT)-MSCs were used due to their ease of maintenance and stable phenotypes, while primary mouse-derived cells were employed to evaluate the effects of modifications on cells most likely to be used in translatable applications. Our studies achieved robust covalent modifications using two distinct covalent conjugation techniques (Figure 1). We sought to employ strategies by which two types of biomolecules, proteins and carbohydrates, could be independently modified in order to evaluate the effects of conjugations to each and facilitate both being used simultaneously to modify different cellular components. The first approach involves direct conjugation of cell surface lysines with reactive NHS esters while the second method employs oxidation of sialic acids followed by reaction with hydrazides to obtain hydrazones. We selected these approaches as they are the most commonly utilized to modify endogenous proteins and carbohydrates, respectively. Both of these methods are amenable to either directly attaching cargo to the cell surface or to facilitate the incorporation of biotin followed by association with avidin protein. Using these methods, we achieved robust covalent chemical modification of cell surfaces. We systematically evaluated the efficiency of surface labeling as well as the viability and migration capabilities of cells labeled with small molecules versus biotin-avidin complexes. Despite prior studies on covalent cell surface modifications for theranostic applications, gaps remain in comparing the impacts of these modifications across cell types. Our findings provide a framework for advancing surface-modified cells as platforms for drug delivery, diagnostics, and regenerative medicine.

## 2. Results and Discussion

### 2.1. Cellular Surface Modifications and Characterization

In order to establish the permissibility of chemical surface modifications (henceforth referred to simply as “modifications”) and their broad effects on cell types frequently used for delivery and other applications, we used both immortalized and primary macrophages (RAW 264.7 and bone marrow-derived macrophages (BMDM), respectively) and stem cells (hTERT and primary MSCs, respectively) in this work. Each cell type was modified using two means of conjugation chemistry, which was used to append two types/sizes of entities. Separately, we used reactions of accessible cell surface amines with NHS-esters (NHS) and oxidation of terminal sialic acids with sodium periodate (NaIO_4_) followed by reactions with hydrazides (Hyd; Figure 1). These two conjugation types were utilized to either attach the small molecule fluorophore fluorescein (FL) or biotin molecules directly to the cell surface. Fluorescein hydrazide was synthesized via the approach shown in Figure S1, Supporting Information. Following biotinylation, avidin-fluorescein isothiocyanate (FITC) was permitted to associate with biotin functionalities, enabling the comparison of small molecules and large (in this case ≈ 70 kDa) entities attached to cells. To summarize, each of the four cell types (RAW 264.7, BMDM, hTERT MSC, and primary MSC), was modified at protein and carbohydrate sites to display FL via direct modification (NHS-FL or Hyd-FL, respectively) or large avidin-FITC proteins via association with cell-conjugated biotin (NHS-B or Hyd-B, respectively).

We performed optimization studies to balance modification extent while maintaining key cellular characteristics such as morphology and viability. For each cell line and modification type, we systematically evaluated incubation temperature(s), labeling time(s), reagent concentration(s), and related parameters, and selected the conditions that resulted in optimal labeling and maintenance of cellular morphology and viability (qualitatively assessed). In this initial study, standardized conditions were used, though further tailoring should be performed for individual cell types and applications. For NHS-based reactions, we found that treatment with either 0.5 mM NHS-FL or 0.5 mM NHS-B followed by 0.05 mg mL^−1^ avidin-FITC for 30 min at 4 °C yielded the best results. Treatments performed with lower concentrations of NHS-FL or avidin-FITC resulted in inconsistent surface labeling while higher concentrations of fluorophore resulted in increased internalization. For hydrazide-based modifications, we found that 1 mM cold NaIO_4_ in PBS (pH 7.4) with 0.5 mM Hyd-FL 0.5 mM or Hyd-biotin followed by 0.05 mg mL^−1^ avidin-FITC yielded optimal modifications. Periodate treatments using lower than 1 mM final concentrations resulted in inconsistent extents of cell labeling, while higher concentrations (10 mM) affected the viability of the cells. Additionally, oxidation steps involving NaIO_4_ were performed at low temperature (4 °C) to limit sodium periodate oxidation to the cell surface and to prevent potential over-oxidation and damage to lipid membranes resulting in cell death.^[49]^

**Figure 2.**
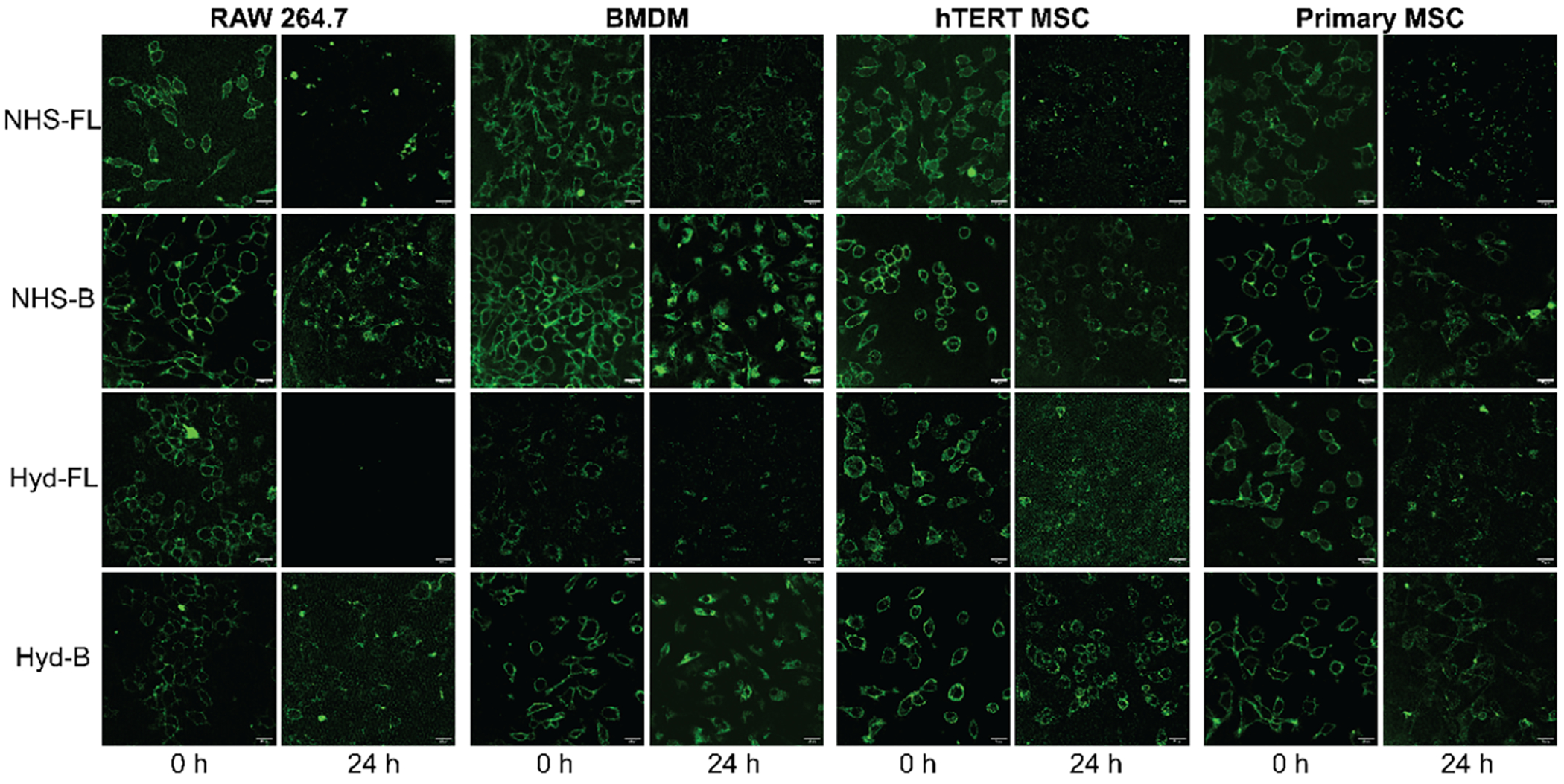
Confocal microscopy data for modification experiments. 0 h and 24 h fluorescent images of NHS and hydrazide modifications performed on all cell types are shown. All images were captured at 60x magnification and scale bars = 20 μM. Respective brightfield and merged images are shown in Figure S2, Supporting Information. Hyd = hydrazide, FL = fluorescein, and B = biotin/avidin-FITC modification.

To qualitatively assess the efficacies of these conjugations, including the distribution of modifications on the cells, confocal microscopy was performed for each cell line and modification, over the course of 48 h (Figure 2, Figure S2, and S3, Supporting Information). To facilitate imaging, cells used in this assay were modified while adhered; for all other studies, cells were modified while suspended (described in the Experimental Section), as typical for other applications. We recognize that different distribution patterns for modifications may be obtained between the two approaches. As expected, the fluorescence observed for each modification and cell type is highest at the 0 and 24 h time-points, with the earlier one showing strong, largely uniform signals throughout the cell surface (Figure 2). In contrast, by the 48 h time-point fluorescence is largely dissipated (Figure S3, Supporting Information). Diminished signals are likely due to a combination of cell division, photobleaching, and internalized fluorophores being sequestered into lysosomes, where they are quenched either due to pH or aggregation (both FL and FITC are pH-sensitive); it is also possible that the probe is being detached. Follow-up studies with cyanine 5 (Cy5, which is not pH-sensitive) are currently in progress,^[50,51]^ and radiotracers and/or mass spectrometry tags can also be used to determine whether the cargo remains associated with the cells. Control experiments confirmed that the modifications occurred through the posited mechanisms for each cell line (Figure S4, Supporting Information). Specifically, no nonspecific association with avidin-FITC was observed in the absence of biotin, and no hydrazide-based strategies resulted in any signals in the absence of sodium periodate-mediated oxidation. We also noted that the morphologies of each of the cell types remained largely consistent following modifications (Figures S2 and S3, Supporting Information).

Overall, modifications utilizing small molecule fluorophores (i.e., NHS-FL or Hyd-FL) appeared to result in substantially more internalization compared to their biotin-based counterparts. Even at the earliest (0 h) time-point, close inspection reveals fluorescence inside of the cells. This is likely because small hydrophobic molecules are readily internalized by cells in comparison to the substantially larger avidin-FITC protein.^[52]^ An outlier in our modification studies seemed to be the Hyd-FL modification in BMDMs, which appeared noticeably dimmer than the other modifications of these cells or in other cells with the same modification. This finding (and others discussed later) indicates that the modifications are to some extent cell line dependent, and differences may be observed. In this instance, we found that a longer incubation time of the Hyd-FL with the cells (120 or 150 min versus 90 min) resulted in a greater extent of labeling, which was then more consistent with the other results (Figure S5, Supporting Information). For the sake of consistency, however, we utilized the 90 min incubation throughout the rest of our experiments, except where otherwise noted.

In addition to the NHS and hydrazide-based modifications, we also examined amine-based modifications using fluorescein amine (NH_2_-FL) and biotin amine (NH_2_-biotin) after oxidizing cell surfaces with NaIO_4_. After incubating with the desired amine, the cells were briefly treated with 0.1 mM cold sodium cyanoborohydride (NaBH_3_CN) in an attempt to reduce the imine bond to a nonlabile amine bond. However, only very low levels of detectable signal were observed after treatments, which were not markedly different from controls where cells were not exposed to oxidant (Figure S6, Supporting Information). This is likely due to the instability of the resulting imine bond, which rapidly hydrolyzes in the aqueous environments that these modifications are performed in.

**Figure 3.**
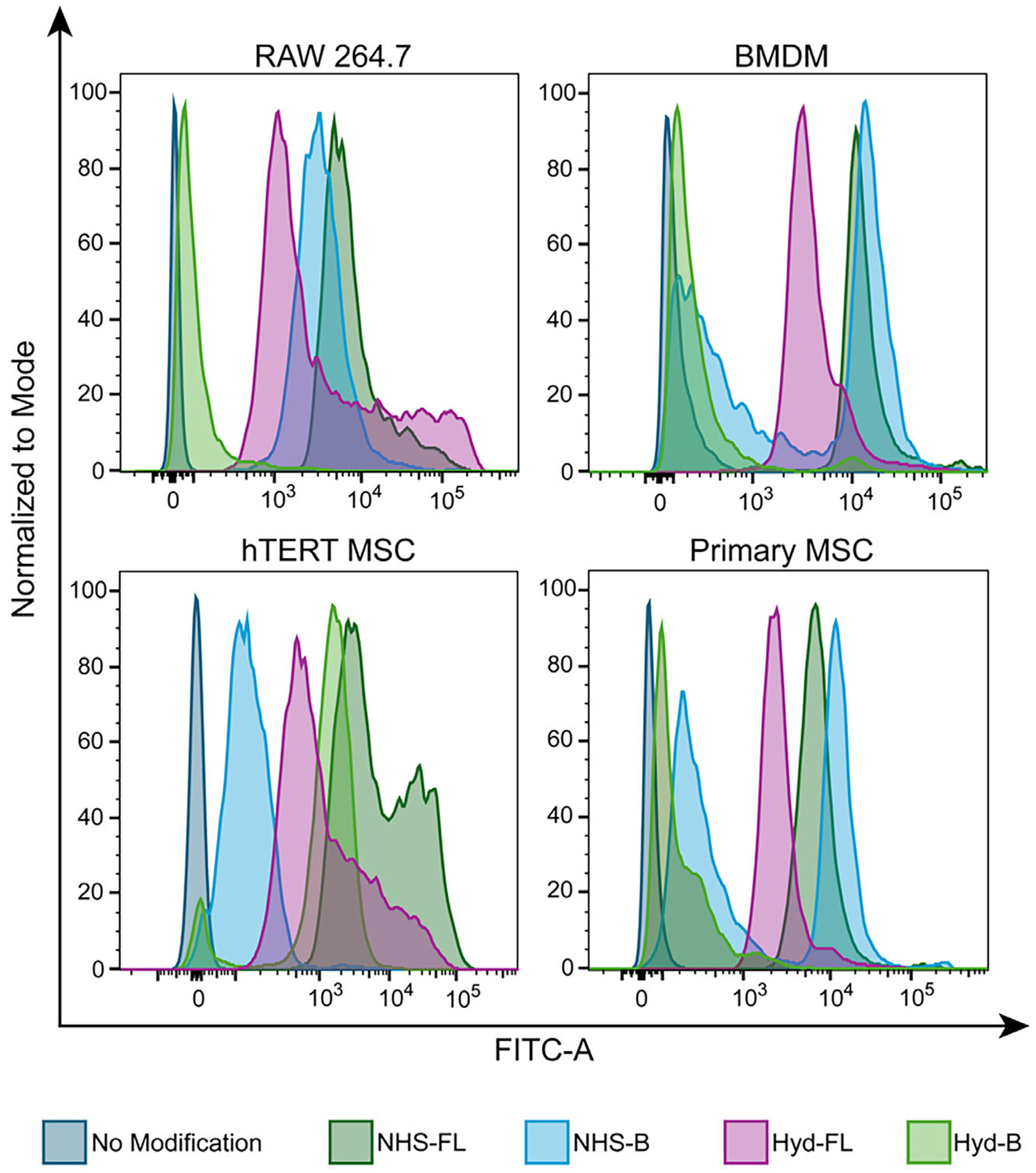
Flow cytometry evaluations of cell modifications. Histograms depict the relative extents of fluorescence for RAW 264.7 (upper-left), BMDM (upper-right), hTERT MSC (lower-left), and primary MSC (lower-right). The color legend is included below and the same for all cell types. The average numbers of cells used per type (independent of modification) were as follows: Primary MSC (8450 cells), RAW 264.7 (6802 cells), hTERT MSC (9032 cells), and BMDM (7984 cells). The exact number of cells per condition used in the figure displayed can be found in Supporting information (Table S1). Hyd = hydrazide, FL = fluorescein, and B = biotin/avidin-FITC modification.

To assess surface modification efficiency and uniformity within each cell type, and capture relative trends across them, we utilized flow cytometry on all four cell types (Figure 3 and Figure S7, Supporting Information). While the patterns observed across cell types were largely similar, this was not uniformly the case. The NHS-FL modification consistently resulted in the highest relative fluorescent signals for each cell type. However, as described above, this may in part be attributed to fluorophore internalization. This finding aligns with the previously discussed confocal microscopy data (Figure 2), where some fluorophore appears to have already been internalized at the 0 h timepoint. The extents of modification were largely uniform within each cell type (evidenced by the presence of a well-defined, single peak), with the exception of hTERT MSCs, where two populations were observed, a major and a minor one appearing as a “shoulder” at a higher intensity. Similarly, the Hyd-FL modification exhibited largely consistent results across all four cell types, and extents of modification were generally lower than for other types. All flow cytometry evaluations of Hyd-FL modifications occurred using the standard, 90 min, incubation time. The Hyd-FL strategy resulted in largely single populations, with similar extents of modification per cell, within the respective cell types, although it resulted in broad distributions for the RAW 264.7 and hTERT MSC cells.

The biotin-mediated modifications showed substantial extents of labeling with greater variability among cell types, resulting in a range of fluorescence intensities. This was particularly the case for NHS-B: while RAW 264.7 and hTERT MSCs exhibited uniform and robust labeling under this condition, in primary cells (BMDMs and primary MSCs) two distinct populations were observed, one showing the highest levels of modification and the other closer to nonmodified controls. This bimodal distribution was unique and was not found with any other modification using standard protocols. The Hyd-B modification resulted in the lowest relative levels of modification across all but the hTERT MSC cell line. We also examined the effects of longer incubation time on the Hyd-B modification (120 min of hydrazide-biotin treatment instead of 90 min), finding that while little changes were observed in the RAW 264.7 and hTERT MSC cell lines, in the BMDMs, a second population emerged with a greater extent of modification (Figure S8, Supporting Information). When comparing trends across different cell surface modification conditions among the four cell types, we observed similar patterns in both primary cell types (BMDMs and primary MSCs), which were also quite similar to those in the RAW 264.7 cells, with the exception that the NHS-B modification yielded only a single population in the latter. Interestingly, the hTERT MSCs appeared to have the most unique trends. While absolute comparisons cannot be made among cell lines, general trends in surface modification efficiency and population heterogeneity observed indicate that they are influenced by cell identity, conjugation type used, and cargo attached.

### 2.2. Effects of Modifications on Cell Viability and Chemotactic Response

**Figure 4.**
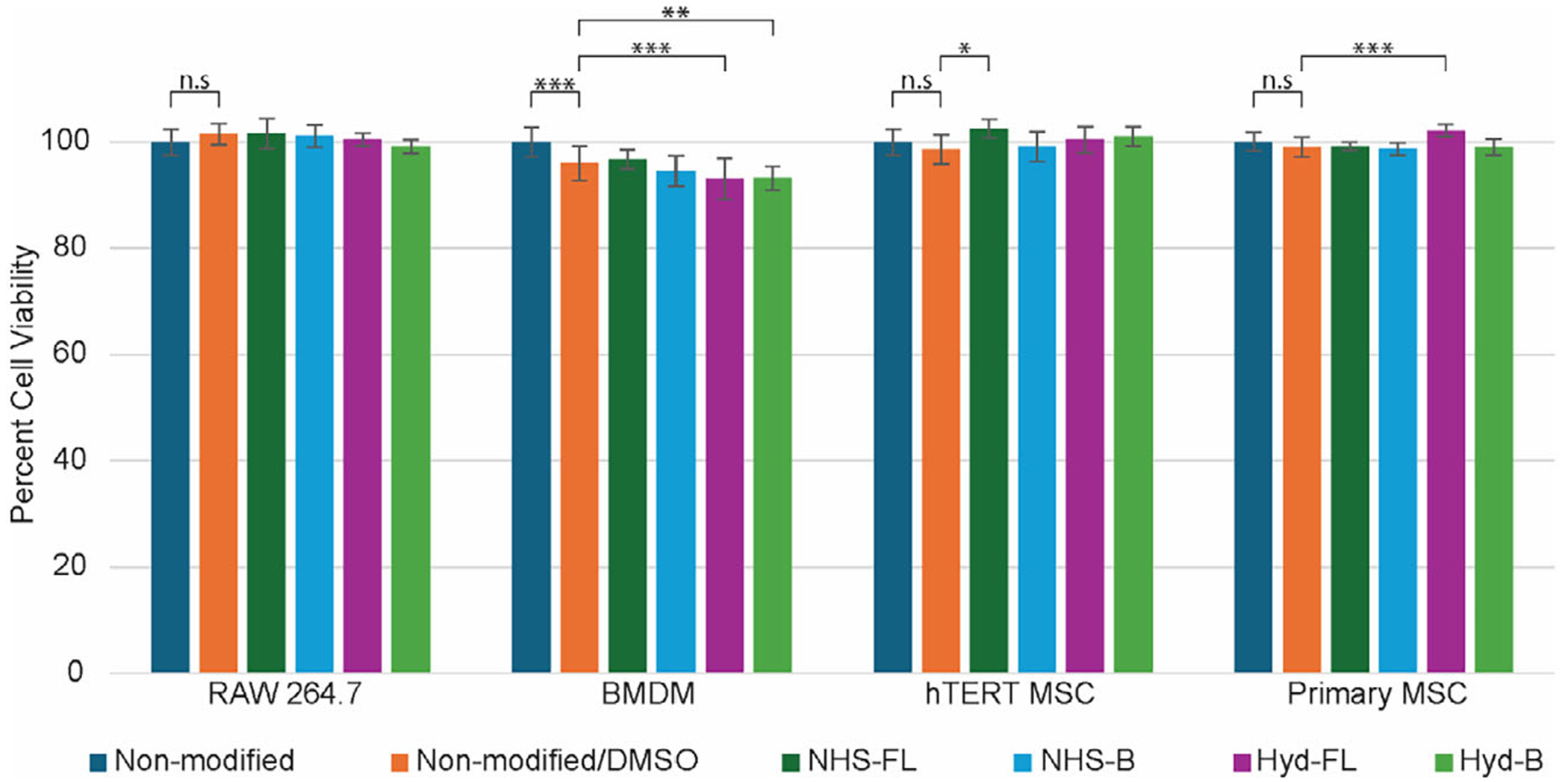
Viability of all cell types 72 h postmodification. Cell viability was performed via Alamar Blue assay. Bars indicate the average cell viability value per modification across 8 biological replicates. Error bars represent standard deviation of the mean. Statistical significance was determined using one-way ANOVA with Dunnett’s correction; n.*s* > 0.05, * ≤ 0.05, ** ≤ 0.01, *** ≤ 0.001. Significance results are shown for all negative control comparisons (nonmodified versus nonmodified DMSO), and otherwise only for significant differences. Nonmodified/DMSO is used as the vehicle control with 0.2% dimethyl sulfoxide (DMSO). Hyd = hydrazide, FL = fluorescein, B = biotin/avidin-FITC modification, and N.S. = not significant.

Cells bearing chemically modified surfaces have tremendous potential to expand fields of research relating to self-regenerating materials, imaging and/or therapeutic entities, and delivery vehicles. Due to the wide scope of areas that can benefit from the development of a surface-modified cell delivery platform, it is critical to ensure that these modification strategies do not impact cellular functions such as viability, morphology, motility, and ability to respond to chemical signals. Hence, we assessed the viability of modified cells via an alamarBlue assay. We observed that our bioconjugation strategies did not impact cellular viability for up to 72 h after initial modifications. Nonmodified cells and nonmodified cells exposed to vehicle (0.2% DMSO in PBS) served as control populations. All modification methods resulted in greater than 90% cell viability in both immortalized and primary macrophages and stem cells (Figure 4). With the exception of BMDM cells, the vehicle controls did not show any significant change in viability compared to the nonmodified cells. For BMDMs, there was a slight reduction in viability for the vehicle control versus the nonmodified cells; the Hyd-FL and Hyd-B cell modifications in this case also resulted in minor, but statistically significant changes versus vehicle. For hTERTs and Primary MSCs there was a slight increase in viability for the NHS-FL and Hyd-FL treatments, respectively. No significant changes were observed across any of the RAW 264.7 samples.

**Figure 5.**
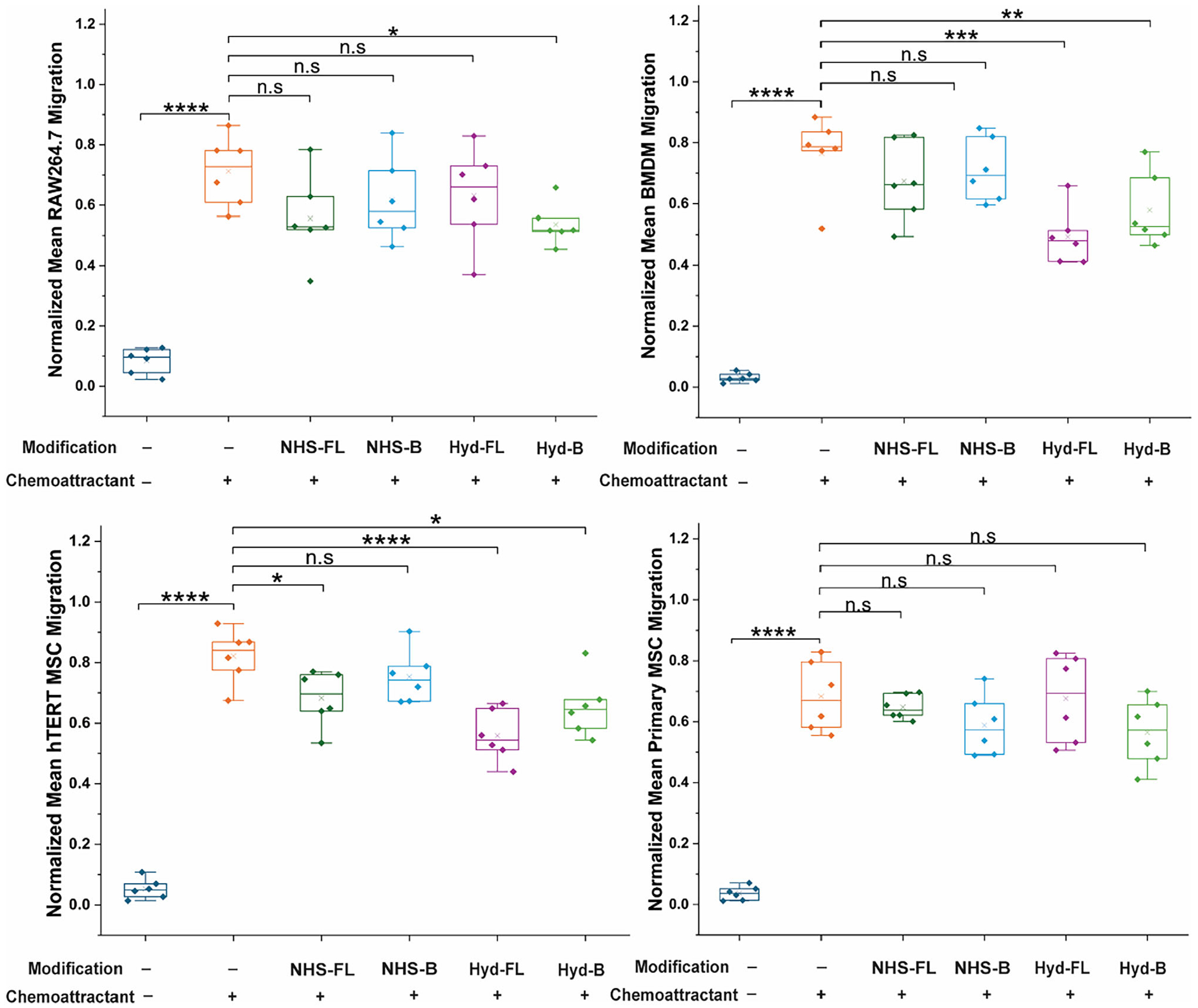
Chemotaxis assessment of modified cells. Cellular chemotactic abilities were determined by using a Boyden chamber assay with MDA-MB-231 conditioned media serving as the chemoattractant and an 8 μm pore membrane. RAW 264.7 (upper-left), BMDM (upper-right), hTERT MSC (lower-left), and primary MSC (lower-right). Nonmodified cells and chemoattractant-lacking controls are indicated by a minus sign (−); presence of chemoattractant is indicated by a plus sign (+). The diamonds in the boxes represent individual data points (*n* = 6) from two independent experiments, the open square represents the mean, and the line bisecting the box represents the median. The bottom and top of the boxes represent the 25th and 75th percentiles respectively, and the whiskers represent the minimum and maximum values. Statistical significance was determined using one-way ANOVA with Dunnett’s correction; n.s. > 0.05, * ≤ 0.05, ** ≤ 0.01, *** ≤ 0.001, **** ≤ 0.0001. Hyd = hydrazide, FL = fluorescein, B = biotin/avidin-FITC modification, and N.S. = not significant.

One of the most advantageous characteristics of certain cell types is their ability to detect chemical signals and traffic toward them, by actively sensing and responding to chemotactic markers and cytokines in particular microenvironments that result in a greater propensity for targeted accumulation.^[53,54]^ Chemoattractants, such as colony stimulating factor (CSF-1), are particularly known to be secreted by wounds, inflamed tissues, and tumors.^[53]^ CSF-1 plays a pivotal role in regulating the survival, proliferation, differentiation, and function of monocytes, and facilitates the recruitment of macrophages and MSCs to tumor sites, areas of inflammation and infection, and wounded tissues.^[53,55]^ The innate homing ability of macrophages and MSCs, along with their capability to cross blood-brain barrier,^[56,57]^ makes them attractive candidates for use and as next generation tools in tissue repair, drug delivery, and theragnostics.^[47,58]^ Since the ability to respond to chemical signals is a pivotal attribute of macrophages and stem cells in many potential applications, we sought to confirm that chemotactic ability of the cells was not impacted post-modification. To this end, we performed a Boyden chamber cell migration assay, counting cells that passed through an 8 μM membrane, to quantitatively measure modified cells’ responses to chemoattractant (Figure 5).^[59]^ We compared the migratory behavior of labeled versus nonlabeled cells in response to MDA-MB-231 cell conditioned media, which served as the chemoattractant in these studies.^[60,61]^ Nonmodified cells not exposed to chemoattractant served as negative controls. Across all four cell types, a significant increase in migration was noticed when comparing nonmodified cells treated with conditioned media to those treated with PBS. Generally, the migration of cells treated with conditioned media was not substantially different between nonmodified and labeled cells. In a few cases (specifically Hyd-B in RAW264.7, BMDM, and hTERT, Hyd-FL in BMDM and hTERTs, and NHS-FL in hTERTs), the changes were found to be statistically significant. It is interesting to note that the hTERT cells and the Hyd-B modifications resulted in the most deviation from the respective positive controls. We surmised that modification types involving more steps may slightly reduce cell migration (Hyd-B has the most of any conjugation used here). However, we were surprised to find this outcome for the hTERTs, since they are an immortalized cell line and their primary (MSC) counterpart did not reveal any statistically significant changes. Nonetheless, the data consistently show that the modified cells retained their ability to migrate and that motility was far more similar to the positive than the negative control in all cases. It is also noted that some degree of variability is common in this assay.^[62]^

## 3. Conclusion and Future Directions

There is an array of applications that exterior-modified cells could benefit, including imaging, diagnostics, and drug delivery. For many purposes, the retention of cellular viability and chemo-sensing/trafficking abilities is equally as important as permissible loading capacities. Hence, we sought to determine if and to what extent covalent bioconjugations could be used to modify immortalized model macrophages and stem cells (RAW 264.7 cells and hTERT MSCs, respectively) and their primary counterparts (BMDMs and primary MSCs). In the work presented here, we demonstrated that the use of bio-compatible cell-surface modifications involving NHS reactions with accessible amines and hydrazide reactions with aldehydes of oxidized sialic acids result in substantial display of cargo and do not impact cellular characteristics vital to their use. We found that not all cell types and modifications yield similar effects, and critically for various applications, the consistency of modification extent within a cellular population can vary, presenting a need for this type of characterization prior to use. The sources of the variations observed among modifications and cell types remain to be uncovered, and should be investigated, as should the fate(s) of the cargo. Similarly, the impacts of chemical modifications on the cells should be evaluated at the molecular level, including transcriptional and translational changes. In future work, the attachment of different types of cargo (e.g., antibodies, peptides, small molecules with varying charges, or nucleic acids) will also be evaluated, both as linkers and payloads. Additionally, since the environment, model, and application where surface-engineered cells are used may also play roles in their efficacy and behavior, it is advisable to evaluate multiple iterations of these tools (e.g., cell type or modification strategy/extent) in the relevant context prior to settling on a single approach. It is also important to note that extents of modification required and tolerated will depend on the specific cargo and application, and should be evaluated on a case-by-case basis.

## 4. Experimental Section

### Materials

Sulfo-NHS-LC-Biotin (**NHS-biotin**) and all other reagents were purchased from Thermo Fisher Scientific except where otherwise noted. Sodium periodate (NaIO_4_) was obtained from Sigma–Aldrich, 5(6)-Carboxyfluorescein NHS (**NHS-FL**) from Biotium, biotin hydrazide (Hyd-biotin) from Thermo Fisher Scientific, biotin hexylamine (**NH_2_-biotin**) from Cayman Chemical, Fluorescein hexylamine (**NH_2_-FL**) from Biotium, and FITC-conjugated avidin from Pierce Thermo Scientific. The synthesis of 5(6)-Carboxyfluorescein hydrazide (**Hyd-FL**) is described in the supporting information.

### Cell Culture

RAW 264.7 and MDA-MB-231 cells were obtained from the American Tissue Culture Collection, hTERT MSCs were obtained from Prof. Shelly Peyton (Chemical and Biological Engineering, Tufts University), and L929 cells were obtained from Prof. Barbara Osborne (Veterinary and Animal Sciences, UMass Amherst). Primary MSCs were isolated from bone marrow of C57BL/6 black mice and primary macrophages were differentiated from the isolated MSCs (described further below). RAW 264.7, MDA-MB-231, L929 cells, primary macrophages, and primary stem cells were cultured in high glucose Dulbecco’s Modified Eagle Medium (DMEM, Gibco) supplemented with 10% fetal bovine serum (FBS, Corning), 1% L-Glutamine (200 mM, L-Glut, Gibco) and 1% antibiotics (100 μg/mL penicillin and 100 μg/mL streptomycin, P/S, Gibco), referred to herein as complete DMEM. hTERT MSCs were cultured in MEM-alpha (Gibco) supplemented with 10% FBS and 1% P/S, referred to as complete MEM-alpha. For detachment from culture flasks/dishes, 0.25% trypsin-EDTA was used with RAW 264.7 and BMDM cells, and 0.05% trypsin-EDTA was used with hTERT MSCs and primary stem cells. All cells were maintained in a humidified 5% CO_2_ atmosphere at 37 °C. Immortalized cells were subcultured approximately once every 3–4 days; cells between passages 7 and 20 were used for all experiments. The differentiated primary macrophages, which are nondividing, were maintained in media that was refreshed every 3–4 days; following isolation, cells were used within three weeks. Primary stem cells were subcultured approximately once every 3–4 days and only cells between passages 3 and 6 were used for experiments.

### Generation of Conditioned Media

Cells were cultured and passaged at least once before being used to generate conditioned media. The procedure used to generate conditioned media follows previously established protocols,^[63–65]^ and was used for both L929 and MDA-MB-231 cells. In brief, cells were cultured in T175 flasks with complete DMEM until they were >90% confluent. At that point, the media was replaced with complete DMEM media and cells were cultured for an additional 7 days. On day 7, the media was collected and filtered through a 0.45 μm syringe filter and stored at −20 °C. For experiments described here, L929 and MDA-MB-231 conditioned media were used within the first six months of being generated.

### Isolation of Primary Mesenchymal Stem Cells and Differentiation into Bone Marrow-Derived Macrophages (BMDMs) from Mice

Isolation of cells from bone (used for both MSCs and BMDMs) was performed following previously established procedures.^[65–68]^ Bones were collected from femurs and tibiae from euthanized C57BL/6 black mice and placed into 0.6 mL microcentrifuge tubes containing a small hole at the bottom (perforated prior to use with an 18G needle). Each 0.6 mL microcentrifuge tube (containing one femur and one tibia) was inserted into a 1.5 mL microcentrifuge tube and centrifuged for 30 s at 10,000 rpm. The 0.6 mL tube with marrowless bones was then discarded.

For isolation of primary MSCs,^[67,68]^ the pelleted bone marrow (in the 1.5 mL-tube) was resuspended in 500 μL of complete DMEM. The contents of each 1.5 mL tube were then transferred to a T75 tissue culture flask containing 5 mL of complete DMEM. The media in these T75 flasks was replaced (with complete DMEM) every 12 h for 72 h. After 72 h, these cells were washed with phosphate-buffered saline (PBS, pH 7.4; Gibco), and the media was replaced every 4 days until the cells were almost confluent (2–3 weeks). At that stage, media was removed, cells washed with PBS, and 0.5 mL of 0.25% Trypsin-EDTA was applied for 2 min at room temperature. The cells that detached within this timeframe were collected, passaged, and cultured in a new T75 flask. To ensure homogeneity, MSCs were passaged at least two times (allowing the cells to become nearly confluent between each passage), and assessed visually.

For differentiation into BMDMs,^[65,66]^ the pelleted bone marrow (in the 1.5 mL tube) was resuspended in 500 μL of 70% complete DMEM and 30% L929-conditioned media, together referred to as differentiation media. The contents of each 1.5 mL tube were then transferred to a T175 tissue culture flask containing 12 mL of differentiation media, and incubated for 3 days, after which the media was replaced with 12 mL of fresh differentiation media for an additional 4 days. Following differentiation, media was replaced with complete DMEM, the cells were assessed visually to ensure no differences in morphology, and they were incubated until used for experiments.

### NHS Modification Protocol

For confocal microscopy imaging experiments involving all cell types except BMDMs, 2.5 × 10^4^ cells in 250 μL of the corresponding complete media were plated in borosilicate glass bottom 8-well chamber slides (Nunc Lab-Tek). For BMDMs, which are nondividing, 5.0 × 10^4^ cells were plated in 250 μL of complete DMEM media. Prior to labeling, the cells were maintained overnight in a humidified 5% CO_2_ atmosphere at 37 °C. The following day, cells were modified while adhered to the chamber slides. First, culture medium was removed, and the cells were washed with 250 μL of PBS. Cells were then incubated with 250 μL of either 0.5 mM **NHS-FL** in 0.2% DMSO in PBS or 0.5 mM **NHS-biotin** in PBS for 30 min at 4°C. After 30 min, the cells were washed three times with 250 μL PBS. For cells treated with NHS-FL, 250 μL of the phenol red free version of the corresponding complete growth media was added and the cells imaged. For cells treated with **NHS-biotin**, 250 μL of 0.05 mg mL^−1^ Avidin-FITC in PBS was then added to the cells and together incubated for 30 min at 4 °C. After 30 min, the cells were washed three times with 250 μL PBS. Following the last PBS wash, 250 μL of the phenol red-free version of the corresponding complete growth media was added and the cells were imaged.

For viability, flow cytometry, and chemotaxis assays, cells were modified while in suspension. For suspension labeling, 3 × 10^6^ cells were isolated per cell type, media removed and washed with 1 mL PBS. Cells were resuspended in 1.5 mL microcentrifuge tubes using 1 mL of the corresponding labeling reagents dissolved in PBS, with all concentrations and incubation times consistent with those used in the adherent labeling protocol described above. For washes, cells were pelleted via centrifugation at 1500 rpm for 5 min and washed with 1 mL PBS 3 times, repelleting after each wash step.

### Hydrazide Modification Protocol

For confocal microscopy imaging experiments involving RAW 264.7, hTERT MSCs, and primary MSCs, 2.5 × 10^4^ cells in 250 μL of the corresponding complete media were plated in borosilicate glass bottom 8-well chamber slides (Nunc Lab-Tek). For experiments involving BMDMs, 5.0 × 10^4^ cells in 250 μL of the corresponding complete media were instead plated in borosilicate glass bottom 8-well chamber slides due to the nondividing nature of these cells. Prior to labeling, the cells were maintained overnight in a humidified 5% CO_2_ atmosphere at 37 °C. The following day, cells were modified while adhered to an 8-well chamber slide. First, culture medium was removed, and the cells were washed with 250 μL of PBS. Cells were then incubated in 250 μL of 1 mM NaIO_4_ in PBS for 30 min at 4°C. After 30 min, the cells were washed three times with 250 μL PBS. Cells were then incubated with 250 μL of either 0.5 mM **Hyd-FL** in 0.2% dimethyl sulfoxide (DMSO) in PBS or 0.5 mM **Hyd-biotin** in 0.2% DMSO in PBS for 90 min at 4 °C. After 90 min, the cells were washed three times with 250 μL PBS. For experiments using longer incubation times, the same conditions were used with the exceptions that cells were incubated for 120 or 150 min (for **Hyd-FL**), and 120 min (for** Hyd-biotin**). For cells treated with **Hyd-FL**, 250 μLof the phenol red free version of the corresponding complete growth media was added and the cells were imaged. For cells treated with **Hyd-biotin**, 250 μL of 0.05 mg mL^−1^ Avidin-FITC in PBS was then added to the cells and incubated for 30 min at 4 °C. After 30 min, the cells were washed three times with 250 μL PBS. Lastly, the PBS wash was removed and 250 μL of the phenol red free version of the corresponding complete growth media was added and the cells were imaged.

For viability, flow cytometry, and chemotaxis assays, cells were modified while in suspension. For suspension labeling, 3 × 10^6^ cells were isolated per cell type, media removed, and washed with 1 mL PBS once. Cells were resuspended in 1.5 mL microcentrifuge tubes with 1 mL of the corresponding labeling reagents in PBS – all concentrations and incubation times were consistent with those used in the adherent labeling protocol described above. For washes, cells were pelleted via centrifugation at 1500 rpm for 5 min and washed with 1 mL PBS three times, repelleting after each wash step.

### Confocal Microscopy

Cell images were acquired using a Nikon Point Scanning C2+ confocal microscope at 60x magnification, with excitation at 488 nm. Cells were imaged at 0, 24, and 48 h timepoints following the completion of modification described above. Between time-points, the cells were incubated at 37 °C under a humidified atmosphere containing 5% CO_2_. Fluorescence, bright-field, and merged cell images were analyzed using Nikon NIS-Elements and FIJI (ImageJ) software Each condition per experiment included 3 biological replicates using cells from the same passage/mouse, modified independently, and 3 fields of view per replicate. At least 2 independent confocal imaging experiments were performed, each using cells from different passages/mice.

### Flow Cytometry

Prior to analyses, the cells underwent the same labeling protocols described above, with the exception that following the final PBS wash step, the cells were resuspended in PBS supplemented with 1% FBS at a density of 1 × 10^6^ cells mL^−1^. The labeled cells were analyzed using a BD LSRFortessa DUAL Flow Cytometry Instrument in the Flow Cytometry Core Facility at the University of Massachusetts Amherst. Data acquisition was performed at a “low” flow rate. The voltage settings for the forward scatter (FSC), side scatter (SSC), and FITC 488 nm (with a 530/30 band pass collection filter) channels for each cell type were set as follows, respectively: RAW 264.7 (FSC: 135 V, SSC: 255 V, FITC: 270 V), BMDM (FSC: 110 V, SSC: 255 V, FITC: 295 V), hTERT MSC (FSC: 125 V, SSC: 240 V, FITC: 207 V), and Primary MSC (FSC: 100 V, SSC: 235 V, FITC: 285 V).

Each sample was gated as shown in the Supporting Information, and singlets were distinguished from doublets by analyzing FSC height (FSC-H) versus FSC area (FSC- A) dot plots (data not shown). Data from each cell type were recorded in triplicate and at least two independent experiments were conducted for each. All data analyses were conducted using FlowJo software (BD Biosciences).

### Viability Assays

Cells were labeled using the suspension labeling protocol described above and immediately plated in 96-well plates at a density of 1 × 10^4^ cells per well. Cells were then incubated in a 5% CO_2_ atmosphere at 37 °C for 72 h. Viability was determined using alamarBlue reagent (Thermo Fisher Scientific), according to the manufacturer’s instructions. Briefly, 10 μL of alamarBlue reagent was added to each well containing cells in the 96-well plate and then allowed to incubate for 4 h in a humidified 5% CO_2_ atmosphere at 37 °C. After 4 h, the absorbance of the wells was measured at 570 nm and 600 nm using a SpectraMax iD3 microplate reader (Molecular Devices). Viability was calculated using the manufacturer’s instructions with the absorbance values obtained. All viability assays were performed independently at least twice, with 8 biological replicates for each assay.

### Chemotaxis Assays

Boyden chamber cell migration assays were performed using transwell inserts (8 μm pore, 6.5 mm, PET membrane, Corning) based on previously established protocols.^[59,69]^ Cells were modified in suspension using the methods described above. A total of 1 × 10^5^ modified cells in 100 μL of corresponding culture media lacking FBS were added to each insert and incubated in MDA-MB-231 conditioned media supplemented with 10% FBS for 18 h at 37 °C, 5% CO_2_. Negative and positive controls (nonmodified cells not exposed to and exposed to chemoattractant, respectively) were also resuspended and added to the inserts in the respective culture media without 10% FBS. Each condition included three biological replicates. After the 18 h incubation, the media was removed, and the inserts were rinsed with PBS. Nonmigratory cells on the upper side of the insert membrane were gently removed using a Q-tip. Migratory cells at the bottom of the insert were fixed in 4% formaldehyde (Pierce 16% with a 4x dilution in PBS), and stained with 10 μg mL^−1^ Hoechst (MilliporeSigma) in Milli-Q water. Membranes were removed from the insert, mounted onto cover-glass slips and imaged using a Nikon Point Scanning C2+ confocal microscope at 405 nm. Cells were counted from four fields of view per membrane, with three membranes per condition using Nikon NIS-Elements and FIJI (ImageJ) software. Box and whisker plots were generated using OriginPro 2017 (Origin lab). All migration assays were performed in at least two independent experiments with three biological replicates per experiment.

## Supplementary Material

Supporting Information

Supporting information for this article is available on the WWW under https://doi.org/10.1002/cbic.202500185

## Data Availability

The data that support the findings of this study are available from the corresponding author upon reasonable request.
