# Supplementary material for: Chemical Surface Modification Tolerance of Primary and Immortalized Macrophages and Stem Cells
Source: Chembiochem. Author manuscript; Available in PMC 2026 Jul 7. (PMC13339997; doi:10.1002/cbic.202500185)
Supplement: Supporting Information [file NIHMS2185953-supplement-Supporting_Information.pdf]

ChemBioChem

Supporting Information

Chemical Surface Modification Tolerance of Primary and Immortalized Macrophages and Stem Cells

Kyle J. Winters, Yacun A. Shen, Emmanuel F. Rivera Iglesias, Jeffrey D. Cullen, Bishnu P. Joshi, Michelle E. Farkas*

Instrumentation

Analytical HPLC data was obtained using a Waters Alliance 2695 Separation Module equipped with a Waters 2487 dual wavelength absorption detector and a Hichrom Apollo 5 µm C18 (250 x 4.6 mm) column. Analyses were performed using Waters MassLynx software. Preparative HPLC was performed using a Teledyne-ISCO ACCQPrep HP150 equipped with a variable UV-VIS 200-800 nm detector and a Teledyne-ISCO RediSep Prep 5 µm C18 (250 x 20 mm) column. Compounds were identified by a Bruker UltrafleXtreme MALDI-TOF/TOF MS.

Synthesis of Hyd-FL

Synthesis of 5(6)-Carboxyfluorescein N-hydroxysuccinimide ester: To a solution of 5(6)-carboxyfluorescein (0.20 g, 0.53 mM, 1 eq) dissolved in 5.00 mL dry N,N-dimethylformamide (DMF) under N_2 was added N,N'-disuccinimidyl carbonate (DSC; 0.55 g, 2.13 mM, 4 eq). The resulting solution was stirred at ambient temperature for 30 min. After time elapsed, triethylamine (Et_3N, 0.93 mL, 5.31 mM, 10 eq) was then added to the solution dropwise and the reaction was allowed to stir at ambient temperature for an additional 24 h. At that time, the solution was used in the next step without further purification. Assessment of the crude mixture via analytical HPLC revealed two new peaks corresponding to the NHS ester isomers, which eluted at 10.02 min and 11.52 min (65:35 H_2O:ACN, 0.1% TFA, isocratic). MALDI-MS cald for $C_{25}H_{15}NO_9$: 473.39, found: 474.227 $(M+H)^+$.

Synthesis of 5(6)-Carboxyfluorescein hydrazide: To the above solution of 5(6)-carboxyfluorescein N-hydroxysuccinimide ester was added hydrazine monohydrate (0.10 mL, 2.13 mM, 4 eq), dropwise. The reaction was then allowed to stir at ambient temperature for 24 h. After 24 h, the solvent was removed *in vacuo* and crude material was purified via prep HPLC (85:15 to 75:25 H_2O:ACN, 0.1% TFA, linear gradient) to yield 42.5 mg of product as a yellow/orange powder (20.49% overall from first step). Analytical HPLC yielded two new peaks corresponding to the two hydrazide isomers eluting at 4.87 min and 7.05 min (75:25 H_2O:ACN, 0.1% TFA isocratic) (98.2%). MALDI-MS cald for $C_{21}H_{14}N_2O_6$: 390.35, found: 391.284 $(M+H)^+$.

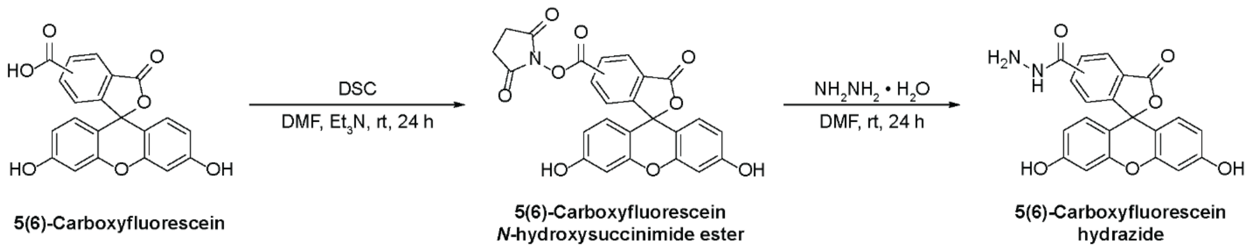

SI Figure 1: Synthetic scheme for generation of 5(6)-carboxyfluorescein hydrazide (referred to as fluorescein hydrazide or Hyd-FL).

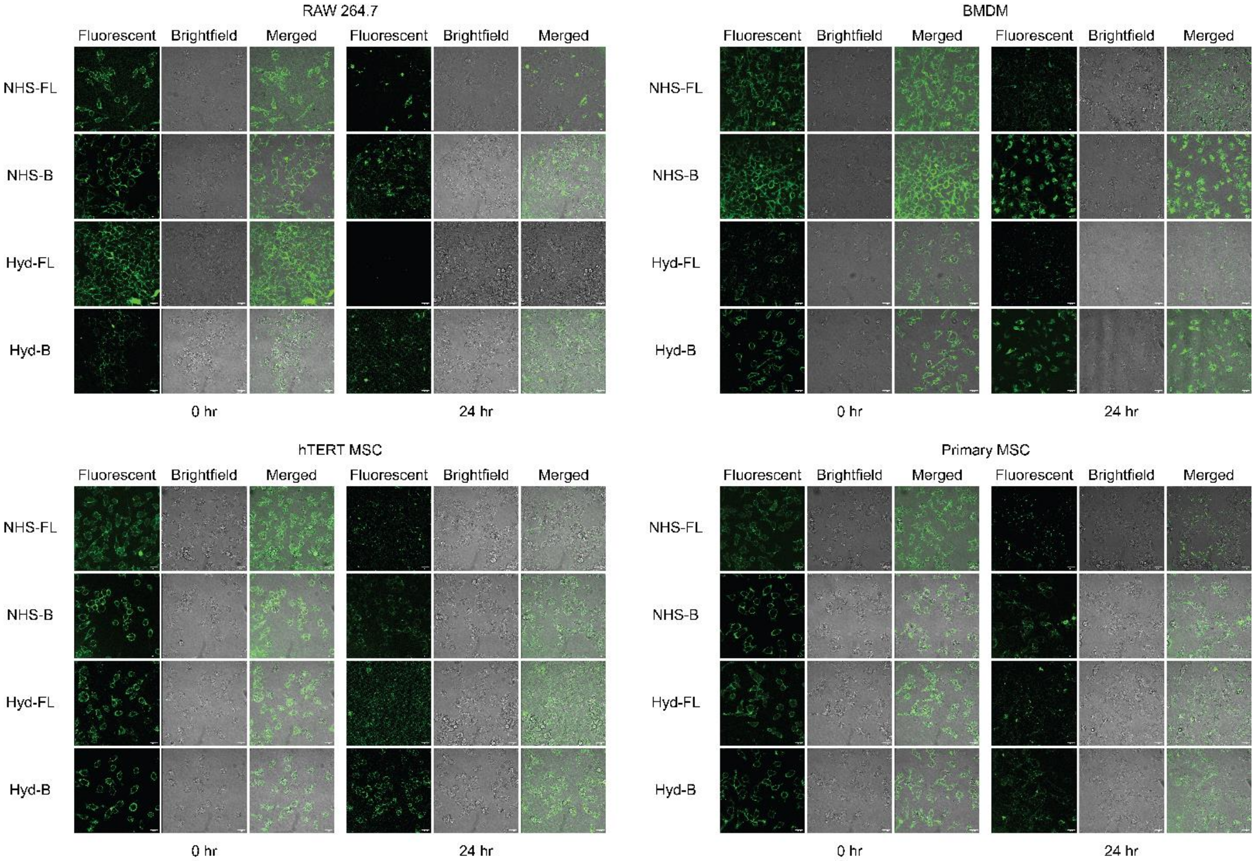

SI Figure 2: Complete confocal microscopy data from labeling experiments at 0 h and 24 h time-points. Fluorescent, brightfield, and merged images are shown following NHS and hydrazide (hyd) modifications performed on all cell types. All images were captured at 60x magnification and scale bars = 20 µM. Hyd = hydrazide, FL = fluorescein, B = biotin/avidin-FITC modification.

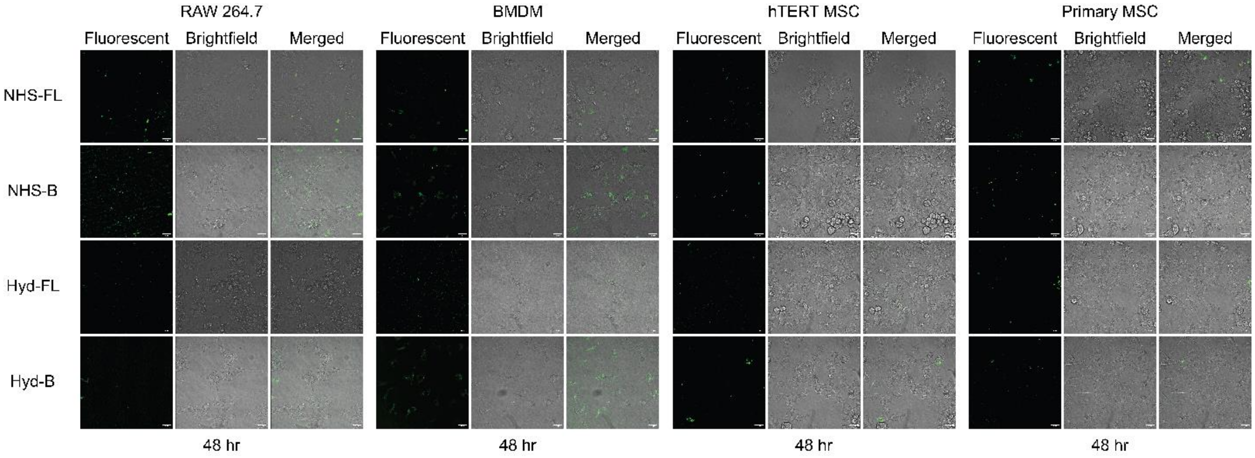

SI Figure 3: Confocal microscopy data from labeling experiments at 48 h time-point. Fluorescent, brightfield, and merged images are shown following NHS and hydrazide (hyd) modifications performed on all cell types. All images were captured at 60x magnification and scale bars = 20 µM. Hyd = hydrazide, FL = fluorescein, B = biotin/avidin-FITC modification.

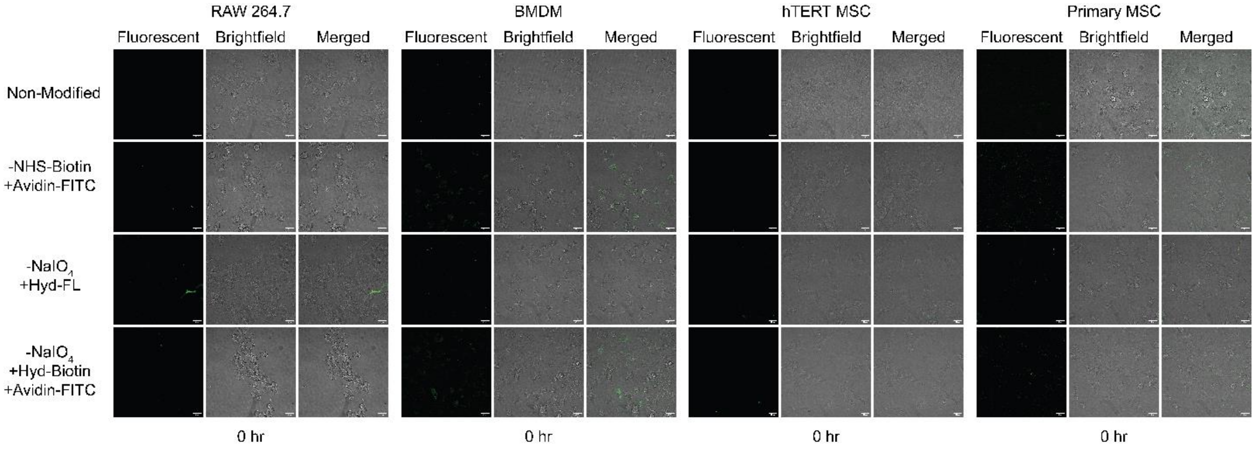

SI Figure 4: Confocal microscopy data from control experiments. 0 h fluorescent, brightfield, and merged images of control experiments performed on all cell types. Exclusion of a reagent during treatment is indicated by a minus sign (-); inclusion of a reagent during treatment is indicated by a plus sign (+). All images were captured at 60x magnification and scale bars = 20 μM. Hyd = hydrazide, FL = fluorescein, B = biotin, $NaIO_4$ = sodium periodate modification.

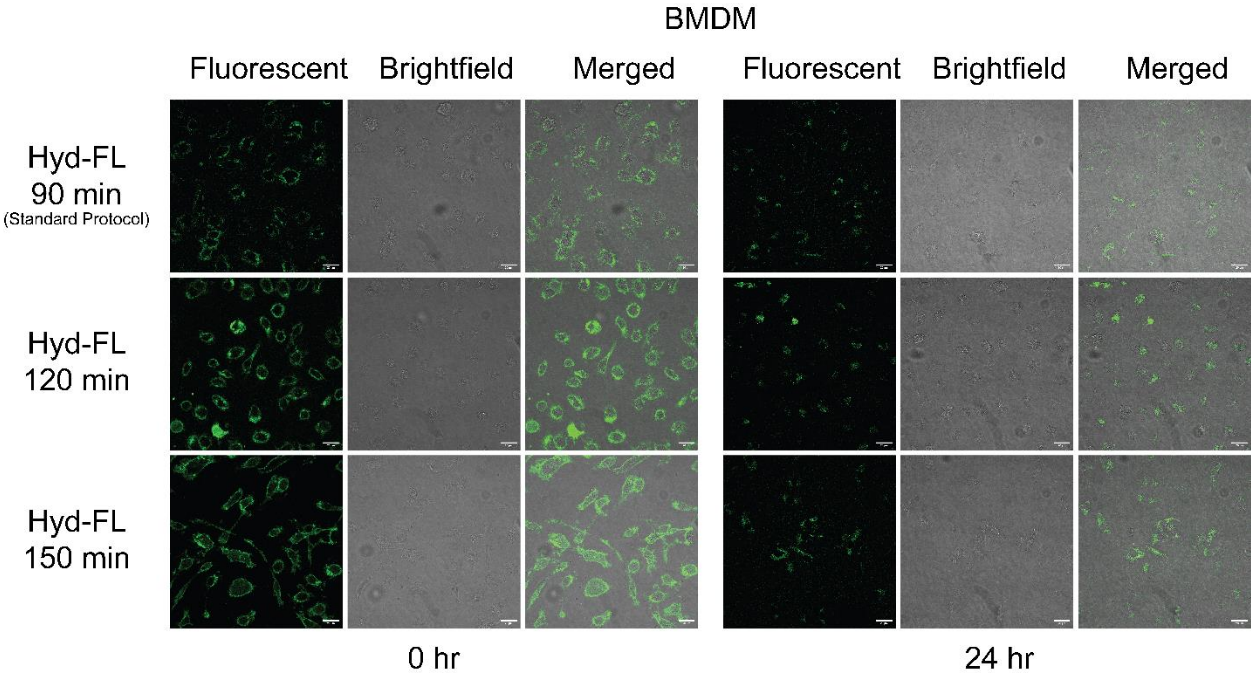

SI Figure 5: Confocal microscopy data from BMDM labeling experiments with extended Hyd-FL incubation times. 0 h and 24 h fluorescent, brightfield, and merged images are shown. Row 1: standard 90 min treatment time resulting in dim fluorescence; Row 2: 120 min treatment time resulting in a greater extent of modification; Row 3: 150 min treatment time resulting in similar results to those observed in row 2. All modifications showed diminished signal at 24 h regardless of treatment time with Hyd-FL reagent. All images were captured at 60x magnification and scale bars = 20 μM. Hyd = hydrazide, FL = fluorescein modification

a.)

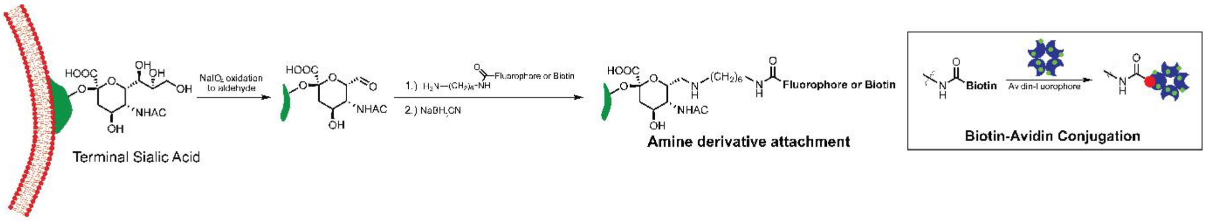

b.)

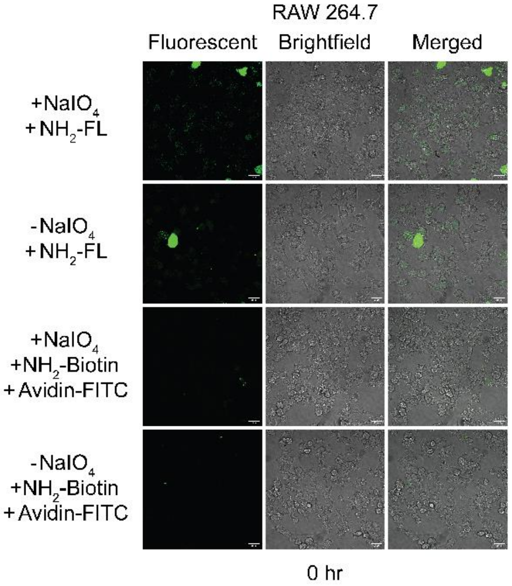

SI Figure 6: Amine-based modifications of cells. **a.)** Overview of strategy used for conjugating amine-bearing cargo to cell surface. Terminal sialic acid residues were oxidized with $NaIO_4$ followed by reaction with desired amine and then reduction via $NaBH_3CN$. These reactions were designed to either directly conjugate fluorophores or biotin moieties to the cell surfaces. Following biotinylation, avidin-FITC can be introduced and associated with surface-conjugated biotins (red orb represents biotin) (inset). **b.)** Confocal microscopy data from labeling experiments. 0 h fluorescent, brightfield, and merged images of amine (NH_2) modifications performed on RAW 264.7 cells. Exclusion of a reagent during treatment is indicated by a minus sign (-); inclusion of a reagent during treatment is indicated by a plus sign (+). All images were captured at 60x magnification and scale bars = 20 µM. NH_2- = amine, FL = fluorescein, B = biotin, $NaIO_4$ = sodium periodate modification.

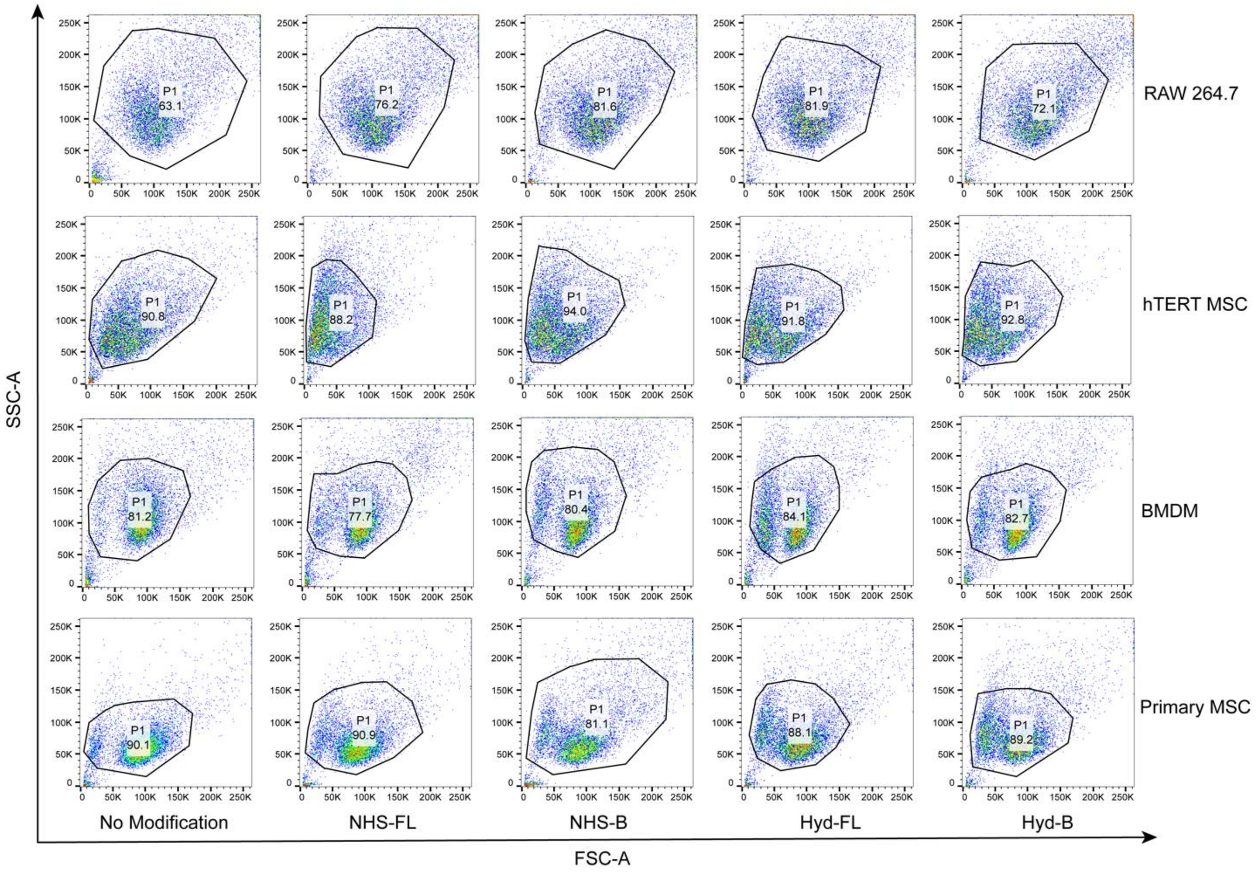

SI Figure 7: Forward/Side scatter plots acquired from flow cytometry experiments (**Fig 3**). FSC-A (x-axis) is indicative of the relative size of the cell and SSC-A (y-axis) is indicative of the granularity of the cell. The enclosed area in each plot represents the cells selected based on the gating parameters. Hyd = hydrazide, FL = fluorescein, B = biotin/avidin-FITC modification.

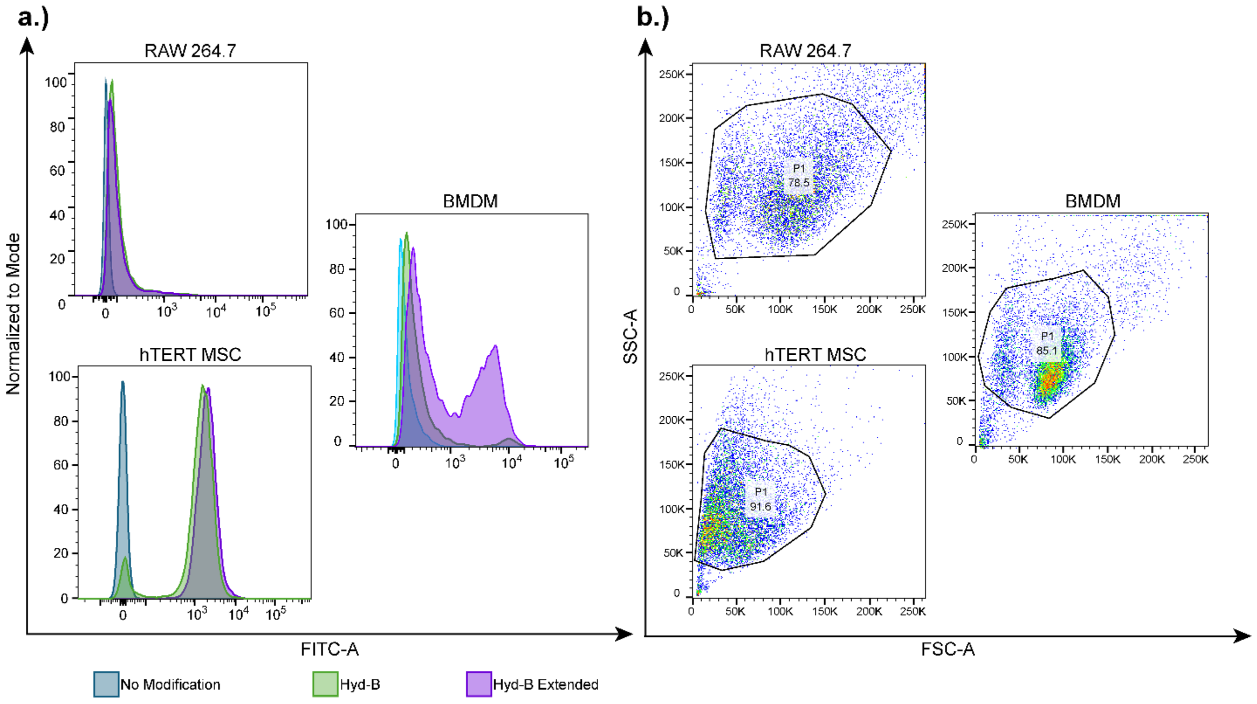

SI Figure 8: Flow cytometry evaluation of cells treated with increased incubation times of Hyd-B. Incubation times were increased to 120 min. instead of the standard 90 min. protocol. **a.)** Histograms depict the relative extents of fluorescence for RAW 264.7 (upper-left), BMDM (middle-right), and hTERT MSC (lower-left). **b.)** The forward and side scatter plots obtained from the flow cytometry experiments are shown for the extended 120-minute incubation time only. Additional gated populations, including those with no modification and with Hyd-B, can be referenced in **Fig S7**. The enclosed area in each plot represents the cells selected based on the gating parameters. Hyd = hydrazide, B = biotin/avidin-FITC modification.

SI Table 1. Post-gating cell counts for each chemical modification and cell type for data shown in **Figure 3**.

Cell Type	Conditions	Cell Count (after gating)
**Primary MSC**	Hyd-B	8793
	Hyd-FL	8625
	NHS-B	7141
	NHS-FL	8776
	No Modification	8913
**RAW 264.7**	Hyd-B	6643
	Hyd-FL	7627
	NHS-B	7367
	NHS-FL	6683
	No Modification	5691
**hTERT-MSC**	Hyd-B	9080
	Hyd-FL	9172
	NHS-B	9292
	NHS-FL	8727
	No Modification	8887
**BMDM**	Hyd-B	8179
	Hyd-FL	8267
	NHS-B	7905
	NHS-FL	7646
	No Modification	7923
